# Supplementary material for: Knowledge, Understanding and Satisfaction with the Implementation of the Performance Management System at a District Hospital in the Madibeng Subdistrict, South Africa
Source: Int J Environ Res Public Health. 2022 Nov 4;19(21):14461. doi: 10.3390/ijerph192114461 (PMC9654986; doi:10.3390/ijerph192114461)
Supplement: Supplementary file 1 [file ijerph-19-14461-s001.zip › ijerph-1991472-supplementary/Supplementary File S1/Questions asked to respondents during the study.pdf]

QUESTIONNAIRE ON PMDS UNDERSTANDING AND SATISFACTION			
**BIOGRAPHIC INFORMATION**			
AGE:			
SEX:			
HIGHEST QUALIFICATION:			
YEARS EMPLOYED AT BDH:			
YEARS EMPLOYED INCL. BDH:			
RANK & JOB TITLE:			
YEARS DOING PMDS AT BDH:			
YEARS DOING PMDS IN INCL. AT BDH:			
Please select one option from the list below for each question OR please rate the items in terms of relevance, whether low medium or high.			
**PMDS QUESTIONS**			
**What is PMDS?**			
A system for managing, developing and rewarding staff			
A phasic system for managing, developing and rewarding staff to achieve the organisation's goals			
An ongoing phasic system for managing, developing and rewarding staff to achieve the organisation's goals			
A system for paying staff bonuses			
**What is the purpose of PMDS contracting?**			
To develop a performance agreement between the supervisor and subordinate			
To agree on how good performance will be rewarded			
The contracting process has no significance and is a formality			
**What is the purpose of a PMDS mid-year review?**			
For the supervisor and official to jointly review the official's performance			
Reach agreement and attach ratings			
Agree on steps to be taken to improve performance			
All of the above			
**What are the PMDS Generic Assessment Factors?**			
The only measures of performance in the PMDS			
Specifi performance standards applicable to all staff			
Elements and Standards to measure Knowledge, Skills and Attributes			
**What are the PMDS Key Responsibility Areas?**			
General performance outputs applicable to all staff irrespective of job description			
key activities that are measurable and are aligned to the employee's job.			
Outputs for senior managers			
**What are the scores for the different PMDS ratings**			
Not Effective			
Very Effective			
Partially Effective			
Fully Effective			
**Satisfaction Level with PMDS contracting process:**			
Reason for your answer:	**Low**	**Medium**	**High**
**Satisfaction level with quarterly review process:**	**Low**	**Medium**	**High**
Reason for your answer:			
**Satisfaction level with final review process:**	**Low**	**Medium**	**High**
Reason for your answer:			
**Satisfaction level with development of staff weaknesses at BDH:**	**Low**	**Medium**	**High**
Reason for your answer:			

**Satisfaction level with recognition of staff strengths at BDH:**	**Low**	**Medium**	**High**
Reason for your answer:			
**Your level of understanding of PMDS:**	**Low**	**Medium**	**High**
Reason for your answer:			
**Your subordinate's level of understanding of PMDS:**	**Low**	**Medium**	**High**
Reason for your answer:			
**Your manager's/supervisor's level of understanding of PMDS:**	**Low**	**Medium**	**High**
Reason for your answer:			
**Satisfaction level with how PMDS disputes are handled at BDH:**	**Low**	**Medium**	**High**
Reason for your answer:			
**Have you ever received PMDS training?**	**Yes**		**No**
**Have you ever been involved in a PMDS dispute/s?**	**Yes**		**No**
**What was/were the cause/s of the dispute/s?**			
Disagreement on scores			
Disagreement of contracting agreement			
Other (Please specify)			
**What is the procedure when there is a PMDS dispute?**			
To follow the normal grievance procedure			
The dispute resolution process outlined in PMDS			
All of the above are applicable			
**What is the aim and purpose of PMDS?**			
To provide a uniform Performance and development system for the Provincial Government			
To provide a uniform performance rewarding system for the Provincial Government			
**What are your recommendations regarding PMDS at BDH?**			
